# Open globe injuries in a croatian cohort: characteristics and prognostic value of the ocular trauma score in predicting the final visual outcome

**DOI:** 10.1186/s40942-025-00696-z

**Published:** 2025-07-01

**Authors:** Ivan Borjan, Ivna Pleština-Borjan, Robert Stanić, Ljubo Znaor, Beáta Éva Petrovski, Goran Petrovski

**Affiliations:** 1 Department of Ophthalmology, University Hospital Center Split, Spinciceva 1, Split, 21000 Croatia; 2 School of Medicine, University of Split, Soltanska 2, Split, 21000 Croatia; 3 Center for Eye Research and Innovative Diagnostics, Department of Ophthalmology, Institute for Clinical Medicine, Faculty of Medicine, Oslo University Hospital, University of Oslo, Postboks, Nydalen, Oslo, 4956, 0424 Norway; 4 UKLONetwork, University St. Kliment Ohridski-Bitola, 7000 Bitola, Macedonia

**Keywords:** Open Globe injury (OGI), Prognostic factors, Ocular trauma score (OTS), Birmingham eye trauma terminology (BETT), Penetrating injury, Globe rupture, Intraocular foreign body (IOFB)

## Abstract

**Background:**

Open globe injuries (OGI) are the most severe and sight-treating ocular traumas encountered in ophthalmology. The purpose of the study is to analyze the characteristics and prognostic factors of OGI and to evaluate the prognostic value of the Ocular Trauma Score (OTS) in predicting final visual outcome.

**Methods:**

A retrospective study analyzing OGI in 56 patients referred to the Eye Clinic, University Hospital Centre Split, Croatia, was conducted between January 2020 and January 2023. The characteristics of OGI and other relevant factors contributing to the final visual outcome were analyzed. The comparison between the final visual outcomes predicted by OTS system and those observed in the study was performed. Non-parametric statistical methods (Wilcoxon, Kruskal-Wallis and chi-square tests) with the Bonferroni correction for multiple comparisons were used in statistical analysis.

**Results:**

The average age of patients was 45 years; 48 (86%) were males and 8 (14%) females. Most of the patients (68%) were admitted to the hospital and treated within 6 h after the injury. Most of the injuries (88%) occurred during leisure time and most often were located in zone 2 (46%). Penetrating injuries were the most frequent injury type (52%), while globe rupture was present in 16 (29%) patients. The posterior segment was affected in 27 (48%) patients. In 16 (29%) patients, final BCVA reached ≥ 0.5. The worst final BCVA– NLP was most commonly experienced in patients with globe rupture (38%). Poor final BCVA was statistically significantly associated with worse initial BCVA (*P* < 0.001), globe rupture (*P* < 0.001), lower OTS category (*P* < 0.001), and the presence of RAPD (*P* < 0.001). The final visual results were comparable with predicted visual outcome by OTS.

**Conclusions:**

The study cohort from Croatia showed that the most important factors in the prognosis of final BCVA in OGI were initial BCVA, injury type, RAPD, localization and extent of the wound, and appropriate, timely surgical treatment in specialized institutions with experienced surgeons. Furthermore, our study findings are mostly consistent with those of the international OTS group and support the view that OTS is a reliable predictor of the final visual outcome.

## Introduction

Open globe injuries (OGI) represent a significant category of ocular trauma, posing a substantial threat to vision and quality of life for affected individuals worldwide. These injuries are defined as full-thickness defects of the eye wall, including the cornea and sclera, resulting from either blunt force trauma or penetrating injuries. The global incidence of OGI is estimated to be between 3.5 and 4.5 per 100,000 population, making them a significant cause of visual morbidity and potential blindness on a global scale [1–4]. According to the World Health Organization (WHO), approximately 59,9 million people suffer annually from eye injuries that restrict daily activities for more than one day, including around 200,000 OGI. Globally, 19 million people experience unilateral blindness or severe visual impairment caused by eye injuries, 2.3 million suffer from bilateral low vision due to trauma, and 1.6 million are bilaterally blind as a result of eye trauma [3–5].

OGI can result from various mechanisms and can be broadly categorized into several types (according to the Birmingham Eye Trauma Terminology– BETT classification): blunt-force-induced globe ruptures, single-entry penetrating wounds, through-and-through perforating injuries, and penetrating injuries complicated by retained intraocular foreign bodies (IOFB), each presenting unique challenges to visual outcomes [6–9].

Developed in 1996 by a group of ophthalmologists led by Dr. Ferenc Kuhn, BETT aims to eliminate ambiguity in the terminology used to categorize ocular trauma, facilitating better communication among healthcare professionals and improving the management of eye injuries [6–9]. BETT encompasses all types of mechanical eye injuries, including both open and closed globe injuries. OGI are categorized by their mechanisms and severity into different types of injury and into three zones based on the anatomical structures involved [6, 8, 9]. By using BETT, ophthalmologists can ensure that they are using consistent language when discussing eye injuries, which is crucial for effective treatment and patient outcomes [6–9].

Despite significant advancements in surgical techniques, imaging modalities, and post-operative management strategies over recent decades, OGI continue to present significant challenges for ophthalmologists worldwide and the prognosis remains uncertain [4, 10–13]. The visual outcomes can range from excellent recovery to permanent vision loss, depending on the nature and extent of the injury.

To predict visual outcomes, Kuhn et al. proposed The Ocular Trauma Score (OTS) to stratify ocular trauma into categories of risk for poor visual prognosis based on characteristics at presentation [14, 15]. The OTS is a widely used tool, which helps in counselling patients and their families about expected visual outcomes and guiding treatment decisions [16].

The management of OGI demands a multifaceted approach, beginning with prompt evaluation and extending through long-term follow-up care. Timely surgical intervention should be performed to restore the structural integrity of the globe, prevent secondary complications, and preserve as much visual function as possible. Post-operative follow-up period is crucial to timely recognize potential complications and to perform secondary interventions as needed, such as vitreoretinal surgery for retinal detachment (RD) or vitreous haemorrhage [17]. In the long-term follow up period, the surgeon should address any chronic complications and optimize visual rehabilitation.

Understanding the epidemiology, classification, and OGI prognostic factors is crucial for developing effective treatment strategies and improving patient outcomes, and this has not been performed in Croatia before.

This paper aims to analyze the characteristics of OGI in a Croatian cohort, and to evaluate the prognostic value of the OTS in predicting final visual outcomes. By performing this analysis, we hope to contribute to the ongoing efforts to enhance the care and visual outcomes of patients with OGI.

## Methods

This is a retrospective study analyzing data from 56 patients with OGI, treated in the Eye Clinic, University Hospital Centre Split, Croatia, between January 2020 and January 2023 (during COVID-19 pandemic). The research was conducted in accordance with the Guidelines of the Helsinki Declaration and received approval from the Research Ethics Committee of the University Hospital Centre Split, Croatia.

For inclusion in the analysis, patients had to be primarily treated in the Eye Clinic, University Hospital Centre Split, and postoperatively followed-up for at least 6 months. Patients with incomplete data were excluded, as well as those who were primarily treated in another ophthalmological institutions and were sent to our clinic for further treatment. Patients with previous injuries or eye diseases that could affect the final visual outcomes were also excluded from the study.

Medical records of all patients were reviewed for age, sex, injury type, localization of wounds and presence of IOFB, admission time, initial and final (after 6 months) BCVA and other factors contributing to the visual outcome (presence of RAPD, endophthalmitis and RD).

Given that the University Hospital Center in Split is the only reference center for OGI treatment in Split-Dalmatia County, we calculated the cumulative and annual incidence rates of OGI during the three-year monitoring period. The calculations were based on data from the 2021 Croatian census.

OGI were meticulously classified and graded using the internationally recognized BETT classification (injury type, injury zone, category of initial and final BCVA) [6].

We analyzed the change in visual acuity 6 months after OGI surgery, by calculating the change in initial BCVA for each patient and determining its significance. The change in BCVA was categorized as: improvement, worsening, or no change. The changes were defined as a shift in initial BCVA from one category to another (to better or worse category) or remaining in the same category. We also investigated the association of all other initial clinical characteristics of OGI (admission time, RAPD, injury type, injury zone and OTS category) with the change in BCVA.

Furthermore, we analyzed the association of initial BCVA and all other OGI characteristics with the final BCVA. Due to the large number of visual categories and the relatively small sample size, we divided the patients into two categories of final BCVA– group with worse BCVA (NLP– 0.095) and group with better BCVA (≥ 0.1), to perform more appropriate statistical analysis.

To assess the prognostic value of the OTS in predicting the final visual outcome, the final BCVA results, obtained 6 months after the injury, were compared with the expected BCVA values predicted by the international ocular trauma scoring system, based on score categories, applied to our data.

To predict the visual outcomes for all patients in the study, the OTS system was utilized. The calculation of the OTS for each patient was performed strictly adhering to the guidelines established by the OTS group (Tables 1 and 2) [14]. The final score categorized the injury into OTS categories from 1 to 5, with higher scores generally indicating a more favorable prognosis for visual recovery.

**Table 1:** Computational method for deriving the OTS

OTS items	Points
A. Initial visual acuity	
1. NLP	60
2. LP to HM	70
3. 0.005 to 0.095	80
4. 0.1 to 0.4	90
5. ≥ 0,5	100
B. Globe rupture	-23
C. Endophthalmitis	-17
D. Perforating injury	-14
E. Retinal detachment (RD)	-11
F. Relative afferent pupillary defect (RAPD)	-10

Adapted from Kuhn F, Maisiak R, Mann L, et al. The Ocular Trauma Score (OTS). Ophthalmology Clinics of North America. 2002;15(2):163–165

**Table 2:** The estimated probability of follow-up visual acuity category at 6 months

		Final visual acuity (%)				
Sum of points	OTS category	NLP	LP– HM	0.05–0.095	0.1–0.4	≥ 0,5
0–44	1	74%	15%	7%	3%	1%
45–46	2	27%	26%	18%	15%	15%
66–80	3	2%	11%	15%	31%	41%
81–91	4	1%	2%	3%	22%	73%
92–100	5	0%	1%	1%	5%	94%

Adapted from Kuhn F, Maisiak R, Mann L, et al. The Ocular Trauma Score (OTS). Ophthalmology Clinics of North America. 2002;15(2):163–165

To ensure a comprehensive evaluation of the predictive accuracy of the OTS, a follow-up period of at least 6 months after primary surgical treatment was implemented for all patients. This extended observation period is crucial as it allows sufficient time for the healing process to stabilize and for any potential complications to manifest. During this follow-up, patients underwent thorough ophthalmic examinations to assess their final visual outcome.

### Statistical analysis

Continuous variables were summarized as medians with ranges (minimum–maximum), and interquartile ranges (IQR), and categorical variables as counts (n) and percentages (%). Normality was assessed using histograms and the Shapiro-Wilk test. Due to the non-normal distribution and small sample size, non-parametric methods were employed.

The Wilcoxon signed-rank test evaluated changes in BCVA over the 6-month. The Kruskal-Wallis test, followed by Dunn’s test with Bonferroni adjustment, compared age and OTS scores across BCVA outcome groups. Associations between categorical variables were analyzed using chi-square (χ²) test or Fisher’s exact test, with post-hoc Z-test for column proportion (Bonferroni-adjusted). The chi-square test also assessed agreement between final BCVA and OTS-predicted outcomes, while the McNemar-Bowker test compared paired categorical BCVA data.

Analyses were conducted using SPSS 24.0 and STATA, with a two-tailed, significance level of 5%.

## Results

The study analyzed data from 56 patients with OGI in observed three-year period. The median age was 46 years (range: 11–85 years). There were 48 (85.7%) males and 8 (14.3%) females (data are not shown). Table 3. presents the clinical characteristics of the study population.

**Table 3:** Distribution of patients in relation to the investigated variables

	*n* (%)
Time from injury incident to surgery (hours)	
≤ 6	38 (67.9)
7–12	2 (3.6)
13–24	9 (16.1)
25–48	3 (5.4)
> 48	4 (7.1)
Injury type	
Globe rupture	16 (28.6)
Penetrant injury	29 (51.8)
Perforative injury	1 (1.8)
IOFB	10 (17.9)
RAPD	
Yes	27 (48.2)
No	29 (51.8)
Injury zone	
1	22 (39.3)
2	26 (46.4)
3	8 (14.3)
Initial BCVA	
NLP	4 (7.1)
LP– HM	25 (44.6)
0.005–0.095	7 (12.5)
0.1–0.4	9 (16.1)
≥ 0.5	11 (19.6)
Final BCVA (after 6 months)	
NLP	7 (12.5)
LP– HM	14 (25.0)
0.005–0.095	4 (7.1)
0.1–0.4	15 (26.8)
≥ 0.5	16 (28.6)
OTS Median (IQR) Range	70 (47–90) 20–100
OTS category	
1	12 (21.4)
2	10 (17.9)
3	15 (26.8)
4	9 (16.1)
5	10 (17.9)

n = number of patients; IOFB = intraocular foreign body; RAPD = relative afferent pupillary defect; zone of injury (according to Birmingham Eye Trauma Terminology– BETT classification of ocular trauma): 1 = trauma involves cornea and limbus; 2 = extending posteriorly from the limbus up to 5 mm into the sclera; 3 = extending posteriorly more than 5 mm beyond the limbus; BCVA = best corrected visual acuity; NLP = no light perception; LP = light perception; HM = hand motion; IQR: Interquartile range; OTS: ocular trauma score; OTS category (sum of raw points): 1 = 0–44; 2 = 45–65; 3 = 66–80; 4 = 81–91; 5 = 92–100

The three-year cumulative incidence of OGI in Split-Dalmatia County was 13.23 (95% CI: 9.76–16.69) per 100,000 inhabitants, yielding an average annual incidence of 4.41 (95% CI: 3.25–5.56) per 100,000 inhabitants (data not shown).

Table 4. shows the association of the investigated variables with changes in BCVA. A statistically significant difference in age was observed between the BCVA-change groups (*P* = 0.027). Post-hoc pairwise comparisons indicated that patients in the improvement group were significantly younger than those in the no change group (*P* = 0.0165) and those in the worsening group (*P* = 0.0096).

Statistically significant changes were demonstrated by comparing the final BCVA with the initial (preoperative) BCVA (*P* = 0.003).

No statistically significant association was found between time from injury to treatment (≤ 6 h vs. > 6 h), presence of RAPD, injury type, injury zone and BCVA-change.

Although statistically significant association was not found between injury type and BCVA-change, the proportion of patients with globe rupture in the group with unchanged BCVA was 3.9 times higher than in the group with BCVA improvement. In contrast, the proportion of patients with IOFB in the group with BCVA improvement was 4.2 times higher than in the group with unchanged BCVA.

A statistically significant association was detected between BCVA-change and OTS category (*P* = 0.006).

**Table 4:** Distribution of patients according to investigated variables in relation to changes in BCVA

	BCVA-change			*P*
	Worsening (*n* = 7)	No change (*n* = 29)	Improvement (*n* = 20)	
Age				
Median (IQR) range	57 (42–77) 21–81	48 (38–56) 11–85	33.5 (27–47.5) 14–77	**0.027**
Time from injury to surgery (hours)				
≤ 6	5 (71.4)	18 (62.1)	15 (75.0)	0.621
> 6	2 (28.6)	11 (37.9)	5 (25.0)	
RAPD				
Yes	3 (42.9)	16 (55.2)	8 (40.0)	0.553
No	4 (57.1)	13 (44.8)	12 (60.0)	
Injury type				
Globe rupture	3 (42.9)	11 (37.9)	2 (10.0)	0.134
Penetrant injury	2 (28.6)	15 (51.7)	12 (60.0)	
Perforative injury	0	1 (3.4)	0	
IOFB	2 (28.6)	2 (6.9)	6 (30.0)	
Injury zone				
1	3 (42.9)	8 (27.6)	11 (55.0)	0.214
2	2 (28.6)	16 (55.2)	8 (40.0)	
3	2 (28.6)	5 (17.2)	1 (5.0)	
OTS category				
1	3 (42.9)	8 (27.6)	1 (5.0)	**0.002**
2	1 (14.3)	5 (17.2)	4 (20.0)	
3	0 (0.0)	7 (24.1)	8 (40.0)	
4	0 (0.0)	2 (6.9)	7 (35.0)	
5	3 (42.9)	7 (24.1)	0 (0.0)	

BCVA = best corrected visual acuity; n = number of patients; IOFB = intraocular foreign body; RAPD = relative afferent pupillary defect; zone of injury (according to Birmingham Eye Trauma Terminology– BETT classification of ocular trauma): 1 = trauma involves cornea and limbus; 2 = extending posteriorly from the limbus up to 5 mm into the sclera; 3 = extending posteriorly more than 5 mm beyond the limbus; OTS category (sum of raw points): 1 = 0–44; 2 = 45–65; 3 = 66–80; 4 = 81–91; 5 = 92–100

Bold results indicate a significance level of *P* < 0.05

Table 5. shows the association of the investigated variables with the final BCVA.

No statistically significant association was found between the time elapsed from injury to surgery, injury zone and final BCVA.

On the other hand, the presence of RAPD, injury type, OTS category and initial BCVA showed a statistically significant relationship with final BCVA (*P* < 0.001).

Post-hoc pairwise comparisons indicated that patients with globe rupture were significantly more likely to have final BCVA of NLP– 0.095 than ≥ 0.1 (*P* < 0.001). Patients with penetrating injury and IOFB were significantly more likely to have a final BCVA of ≥ 0.1 than NLP– 0.095 (*P* < 0.001 for both). No significant difference between the NLP– 0.095 and ≥ 0.1 groups was observed for perforative injuries (*P* = 1.000).

**Table 5:** Distribution of patients according to investigated variables in relation to the final BCVA

	Final BCVA		P*
	NLP– 0.095 (*n* = 25)	≥ 0.1 (*n* = 31)	
Time from injury to surgery (hours)			
≤ 6	20 (80.0)	18 (58.1)	0.086
> 6	5 (20.0)	13 (41.9)	
RAPD			
Yes	21 (84.0)	6 (19.4)	**< 0.001**
No	4 (16.0)	25 (80.6)	
Type of injury			
Globe rupture	15 (60.0) ^a^	1 (3.2) ^b^	**< 0.001**
Penetrant injury	9 (36.0) ^a^	20 (64.6) ^b^	
Perforative injury	0 (0.00) ^a^	1 (3.2) ^a^	
IOFB	1 (4.0) ^a^	9 (29.0) ^b^	
Injury zone			
1	8 (32.0)	14 (45.2)	0.660
2	13 (52.0)	13 (41.9)	
3	4 (16.0)	4 (12.9)	
OTS category			
1	12 (48.0) ^a^	0 (0.0) ^b^	**< 0.001**
2	6 (24.0) ^a^	4 (12.9) ^a^	
3	7 (28.0) ^a^	8 (25.8) ^a^	
4	0 (0.0) ^a^	9 (29.0) ^b^	
5	0 (0.0) ^a^	10 (32.3) ^b^	
Initial BCVA			
NLP	4 (16.0) ^a^	0 (0.0) ^b^	**< 0.001**
LP– HM	19 (76.0) ^a^	6 (19.4) ^b^	
0.005–0.095	2 (8.0) ^a^	5 (16.1) ^a^	
0.1–0.4	0 (0.0) ^a^	9 (29.0) ^a^	
≥ 0.5	0 (0.0) ^a^	11 (35.5) ^b^	

BCVA = best corrected visual acuity; n = number of patients; IOFB = intraocular foreign body; RAPD = relative afferent pupillary defect; zone of injury (according to Birmingham Eye Trauma Terminology– BETT classification of ocular trauma): 1 = trauma involves cornea and limbus; 2 = extending posteriorly from the limbus up to 5 mm into the sclera; 3 = extending posteriorly more than 5 mm beyond the limbus; OTS category (sum of raw points): 1 = 0–44; 2 = 45–65; 3 = 66–80; 4 = 81–91; 5 = 92–100

Same letters (a, a) indicate no significant difference between the groups. Different letters (a or b) indicate a significant difference between the groups; *P = P-value: after applying the Bonferroni correction; bold results indicate a significance level of *P* < 0.05

**Fig. 1:**
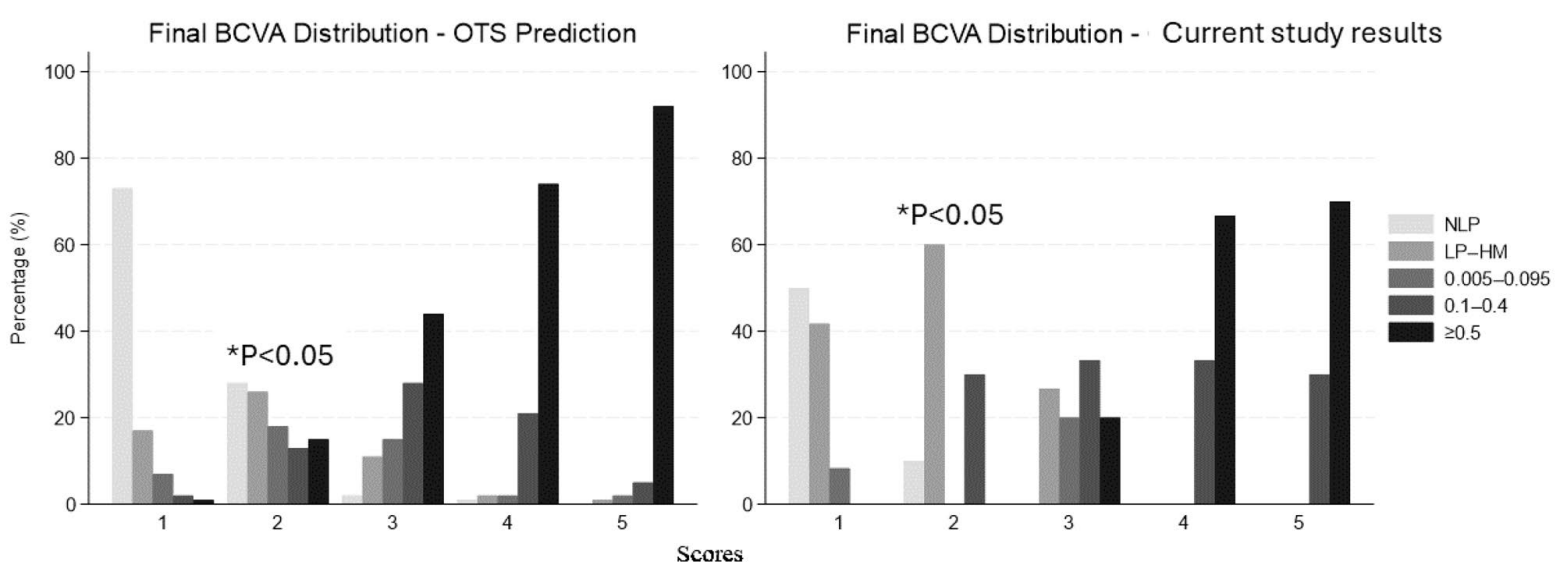
Comparison of final BCVA outcomes between current study and OTS predictions across different score categories. Scores = OTS categories (sum of raw points): 1 = 0–44; 2 = 45–65; 3 = 66–80; 4 = 81–91; 5 = 92–100; NLP = no light perception; LP = light perception; HM = hand motions; OTS = ocular trauma score; A statistically significant difference was found in OTS category 2 (*P* = 0.023)

Figure 1. shows a side-by-side comparison of the final BCVA outcomes between the study results and OTS predictions across five OTS categories. Each category reflects increasing severity of trauma (1 being the most severe and 5 the least). Each pair of bars (one for OTS predictions, one for the current study results) allows for a direct comparison of expected vs. observed visual outcomes within that score category. A statistically significant difference was observed in OTS category 2 (*P* = 0.023), where our study had significantly fewer number of patients within NLP and ≥ 0.5 final BCVA groups, compared to OTS prediction, and higher proportion of patients within LP– HM (60% vs. 26%) and 0.1–0.4 (30% vs. 13%) final BCVA groups. Furthermore, in our study no patients in OTS category 2 achieved a good visual outcome (≥ 0.5), whereas OTS predictions suggested 15% would, though this did not reach statistical significance after Bonferroni correction. For all other categories, the distributions between predicted and observed outcomes were not significantly different.

## Discussion

This retrospective study analyzed the characteristics of OGI in 56 patients in a Croatian cohort (focusing on the factors associated with final BCVA) and evaluated the prognostic value of the OTS in predicting final visual outcomes. OGI are significant cause of permanent visual impairment and visual loss globally [10, 18, 19]. Although the incidence rate of OGI is relatively low the consequences can be devastating [1–5, 20]. Our findings offer valuable insights into the epidemiology and visual prognosis of these severe ocular traumas in Croatia.

The global incidence of OGI varies significantly across regions and populations, influenced by socioeconomic, occupational, demographic (age, gender), and geographic factors. High-income countries report lower OGI incidence rates (3–4.49 per 100,000 in the USA and Germany) compared to certain part of China, where rates can reach up to 27.7 per 100,000 [2, 19, 21]. Our results (annual incidence of 4.41 per 100,000) are consistent with the incidence of OGI in developed countries [2, 3].

Numerous studies highlight age and gender differences in the incidence of OGI [10, 12, 17–19, 22–24]. Men account for approximately 75–85% of cases and tend to be younger at the time of injury. Our research also revealed a higher incidence of OGI in men (85.7%) compared to women (14.3%), with a male-to-female ratio of approximately 6:1. The median age was 46 years and is 33 years lower in men than in women. Other studies have shown similar average ages [22–24]. The mechanisms of injury also differ by gender. Men are more likely to sustain penetrating injuries, whereas women often experience blunt globe ruptures, commonly due to falls [25]. Women generally present with worse initial visual acuity and may have poorer final outcomes [26]. In our study, globe ruptures also emerged as the predominant injury type among women (75.0% of cases), while men predominantly sustained penetrating injuries (56.3% of cases). Men are consistently shown to be at higher risk for ocular trauma, likely due to increased participation in occupational and recreational activities associated with potential eye hazards [3, 10, 12, 14, 17–19, 22–24, 27–29].

The high proportion of ocular injuries in our study had occurred during leisure activities (88%) which can be largely attributed to the COVID-19 pandemic at that time and is notably higher than typically reported in pre-pandemic studies [30]. The COVID-19 pandemic significantly altered daily routines and activity patterns. Lockdowns, social distancing measures, and restrictions on workplace operations likely shifted individuals toward home-based activities [31]. Additionally, the closure of gyms, organized sports facilities, and reduced access to professional services may have contributed to an increase in unsupervised or improperly equipped leisure activities, further elevating the risk of injury.

As supported by the literature, visual recovery in patients with OGI varies significantly and is influenced by several key factors, including type of injury and its mechanism, initial BCVA, presence of RAPD, location and extent of wound, presence and nature of IOFB, time elapsed from injury to treatment, age and the presence of complications such as endophthalmitis or RD [8, 10, 13, 14, 19, 22, 32–34].

The study analysed the association of various clinical variables with changes in BCVA following OGI. Statistically significant changes were observed when comparing initial BCVA to final BCVA (*P* = 0.003). While BCVA improved in 35.7% of patients, it deteriorated in 12.5%, and it remained unchanged in 51.8%.

In our study, the time elapsed from injury to treatment did not show a statistically significant association with changes in BCVA. The reason for this could be the fact that 67.9% of patients were admitted and treated within 6 h after the injury and 87.5% within the optimal 24 h window. This suggests efficient triage and referral systems in our region in Croatia, which is critical for optimizing outcomes in OGI. This is in line with studies that emphasize the importance of early surgical intervention in improving visual prognosis and minimizing severe complications like endophthalmitis [35, 36]. Although is true that prompt repair is crucial, the exact timing within the first 24 h may not significantly affect final visual acuity [4, 37]. In accordance with the above, another study, which was conducted at our department during the Homeland War in Croatia, showed a statistically significant correlation between the time elapsed from injury to surgery. However, in this group of patients the differences in time from injury to surgery were significantly greater compared to this study (from less than 6 h to over a month) [36].

The distribution of injury types in our study, revealed penetrating injuries as the most frequent (51.8%), followed by globe rupture (28.6%) and only one case of perforating injury. This distribution is consistent with reports from other trauma centres [21, 38]. Penetrating injuries from sharp objects can cause significant intraocular damage, while globe ruptures lead to even worse visual outcomes due to extensive structural damage [39]. However, penetrating injuries have a better prognosis than perforating injuries, especially if the wound is anterior located and small [4, 19, 38]. The involvement of the posterior segment in 48.2% of patients in our study indicates the severity of these injuries and the potential for long-term visual impairment. Globe ruptures were more prevalent among patients with no improvement in BCVA, while penetrating injuries and IOFB were associated with better BCVA outcomes. This finding emphasizes the importance of injury type in predicting visual prognosis. The results are consistent with the results of other studies [4, 10, 19, 38].

The zone of injury did not show a statistically significant association with changes in BCVA in our study (*P* = 0.214). However, injuries involving zone 1 (cornea and limbus) were more likely to result in improved BCVA compared to posterior injuries involving zones 2 and 3. Injuries in zone 3 are generally associated with poorer outcomes due to their posterior location and potential for severe complications [20, 40].

The presence of RAPD was shown to be statistically significantly associated with final BCVA (*P* < 0.001); patients with RAPD were more likely to have a worse visual outcome compared to those without. Presence of RAPD is indicative of severe optic nerve and retinal damage and is strongly associated with poor visual outcome [10, 41]. In a Singapore-based study, more than 50% of OGI patients with RAPD had final BCVA worse than HM [10]. According to Rahman et al., 48% of eyes with RAPD were enucleated, which corelates with the results of Peramici et al. (55% eyes with RAPD were enucleated) [42, 43].

Age was significantly associated with changes in BCVA (*P* = 0.027). Younger patients demonstrated greater improvement in BCVA compared to older patients, suggesting that age-related factors such as healing capacity may influence recovery. Our findings are supported by number of published evidence indicating that younger patients tend to have better final BCVA and a higher likelihood of excellent visual recovery [11, 13, 37]. These studies interpret the association between younger age and better visual recovery by a great healing potential (better tissue regenerative capacity, faster wound healing and better vascular supply). Therefore, age is considered as an independent prognostic factor for final visual outcome. The lack of data on pre-injury eye condition and visual acuity could be a limitation when interpreting whether the better final BCVA in young patients is due to age or some other factors. However, in our study we specifically excluded patients with a history of previous ocular trauma, prior ocular surgery, or other pre-existing eye diseases. By applying these exclusion criteria, we aimed to minimize the likelihood that patients had significant visual impairment before the injury, thereby reducing potential confounding effects on the final BCVA outcomes.

Initial BCVA at presentation was strongly associated with final visual outcome (*P* < 0.001). The results corroborate the findings of the original OTS study by Kuhn et al. and other validation studies [10, 14, 20, 40]. This highlights the importance of prompt and accurate assessment of BCVA in patients with OGI. In our study, the final BCVA of ≥ 0.5 was achieved in 28.6% of patients, which is comparable or slightly better to outcomes reported in other OGI studies [22, 38, 40].

The development of endophthalmitis in 2 patients (3.6%) highlights the persistent risk of infection in OGI, despite prophylactic antibiotic treatment, and underscores the need for vigilance in post-operative care. The rate of endophthalmitis in our study is comparable to the rates reported in other large OGI series. However, the frequency of endophthalmitis following OGI varies widely across studies, from 1.0 to 11.9% or even higher depending on risk factors influenced by injury characteristics (e.g., presence and nature of IOFB), geographic factors, and prophylactic measures [44–47].

Our study mainly reinforces the utility of the OTS as a valuable prognostic tool in OGI. The final visual results were compatible with the predicted visual outcome by OTS (with exception for OTS category 2) supporting its reliability in clinical practice. This information is invaluable for counselling patients and guiding treatment decisions.

This study demonstrated fewer patients with final BCVA of NLP and ≥ 0.5, and significantly higher proportion of patients with LP– HM (60% vs. 26%) and 0.1–0.4 (30% vs. 13%) compared to OTS visual predictions. The shift toward intermediate visual acuity outcomes may reflect reduced heterogeneity within OTS category 2 in our population, where injuries were severe enough to prevent excellent outcomes (≥ 0.5) but not severe enough to result in complete blindness (NLP). Early surgical intervention in our patient cohort (67% of injuries were treated within 6 h, and 88% within 24 h) and modern surgical techniques likely mitigated severe complications (e.g., endophthalmitis, RD) and contributed to the reduced incidence of NLP outcomes. Conversely, injury severity and an effort to save every eye, even the most severely damaged one, may have increased the incidence of LP– HM. Conventional OTS has been given at that time, when the enucleation was preferred practice in severe trauma for fear of sympathetic ophthalmitis. Today, enucleation rate is decreased as better treatment modalities are available [12, 13, 47].

Several studies have validated the OTS as a reliable predictor of visual outcomes in OGI with a reported accuracy of correctly predicting outcomes within one visual category in about 77% of cases [10–13, 20, 47]. However, some studies have also identified limitations of the OTS, particularly in predicting outcomes in lower (severe) OTS categories and in specific subgroups of patients, such as those with severe posterior segment injuries [10, 16, 20, 48].

Although the OTS is a validated tool with high predictive capability for visual outcomes following OGI repair, our findings highlight discrepancies in category 2 outcomes, which suggest that modern surgical advancements and evolving in recognition of epidemiological factors may necessitate recalibration of the OTS. That emphasises the need to explore incorporating additional variables (e.g., macular status, time-to-surgery) to enhance its predictive accuracy. These insights underscore the importance of updating prognostic tools to better reflect clinical realities and improve individualized outcome predictions.

There is a limited body of research on OGI originating from Croatia and neighboring countries [36, 49, 50]. Comparisons with our findings are limited due to differences in study design, population characteristics (some studies were focused on paediatric population, while one was conducted during the Croatian Homeland War), time periods and the fact that OTS was not calculated in the most of these studies. Therefore, our results are more readily comparable to findings reported in cited studies (outside our region).

Our study has several limitations. First, the retrospective design introduces the potential for selection bias and data inaccuracies. Second, the relatively small sample size may limit the generalizability of our findings. Third, the study was conducted at a single centre, which may not be representative of all OGI populations. Fourth, we did not analyse the impact of specific surgical techniques or post-operative management strategies on visual outcomes.

Although the number of cases in our study is relatively small, its strength lies in the inclusion of only patients with OGI who had complete data required for calculating OTS according to the international guidelines, as well as a minimum follow-up period of 6 months after the injury to determine final visual acuity. In contrast, some studies report incomplete data (often characterized as “0”) and shorter post-operative follow-up periods [47]. Additionally, we excluded patients with potentially confounding factors for final visual outcomes (e.g., other diseases or previous trauma) to enhance the study’s credibility. Therefore, we believe that the mentioned limitations do not diminish the significance of our findings.

Future research should focus on addressing these limitations by conducting prospective, multi-centre studies with larger sample sizes. Those studies should also investigate the impact of specific interventions, including advanced surgical techniques and novel therapeutic agents, on visual outcomes in OGI.

## Conclusions

This study highlights the significant impact of OGI on visual outcomes and the importance of timely and appropriate management. Initial BCVA, injury type, wound location and extent, timely surgical intervention and RAPD were identified as critical prognostic factors. The devastating impact of OGI on vision, coupled with the fact that these injuries are largely preventable, underscores the need for ongoing efforts to prevent them through public health strategies and also to investigate novel surgical techniques. Although our study showed a generally high concordance between observed visual outcomes and those predicted by the OTS system, it emphasizes the need for regular revalidation of this system to enhance its prognostic value.

## Data Availability

No datasets were generated or analysed during the current study.
